# Supplementary material for: Transcriptomic Insights into Mechanisms of Early Seed Maturation in the Garden Pea (Pisum sativum L.)
Source: Cells. 2020 Mar 23;9(3):779. doi: 10.3390/cells9030779 (PMC7140803; doi:10.3390/cells9030779)
Supplement: Supplementary file 1 [file cells-09-00779-s001.zip › Supplementary materials/FileS4.zip!/FileS3/TFs - Results/Supporting Maps&Profiles/Topology Profiles.pdf]

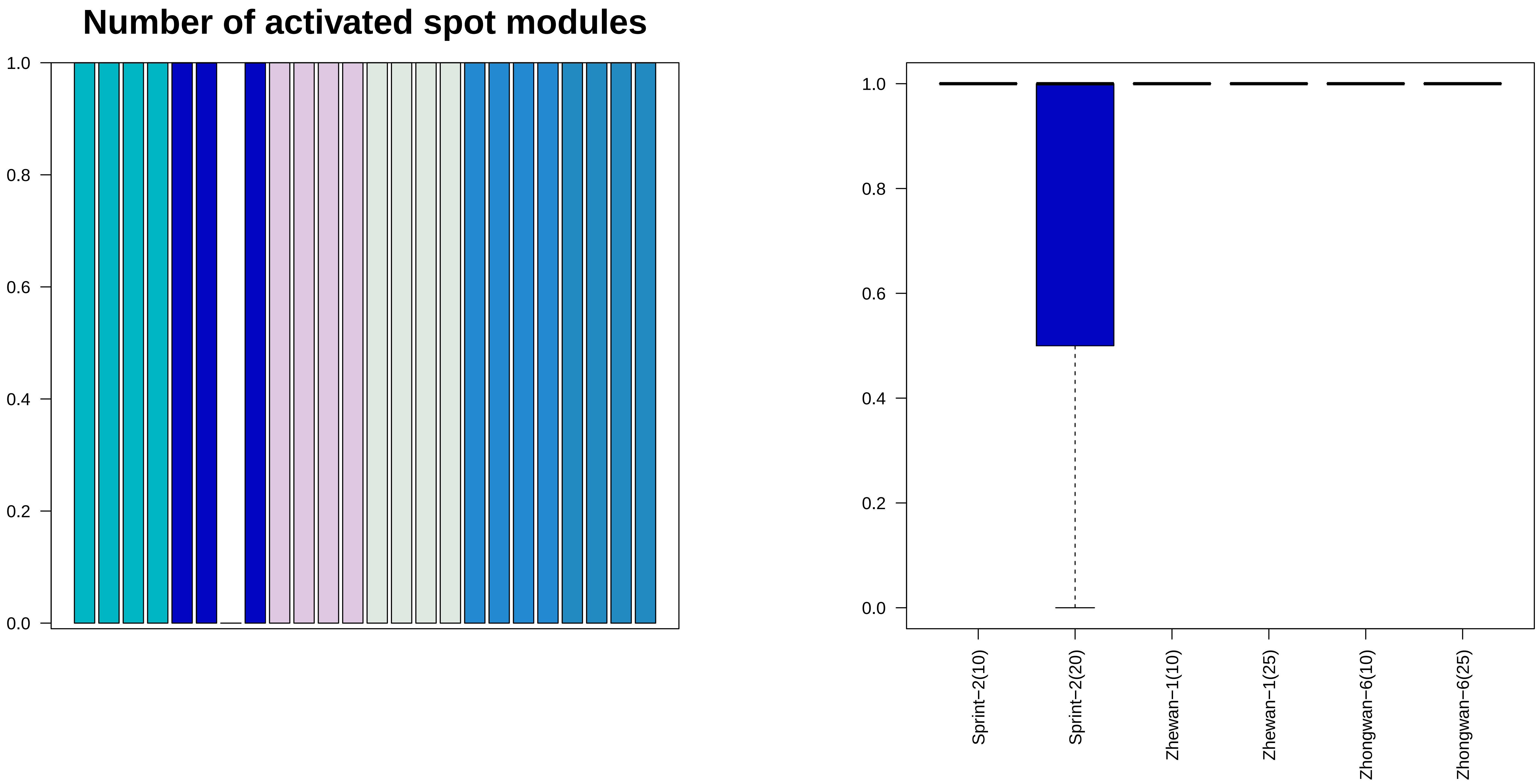

Number of activated spot modules
0.0
0.2
0.4
0.6
0.8
1.0
Sprint−2(10)
Sprint−2(20)
Zhewan−1(10)
Zhewan−1(25)
Zhongwan−6(10)
Zhongwan−6(25)

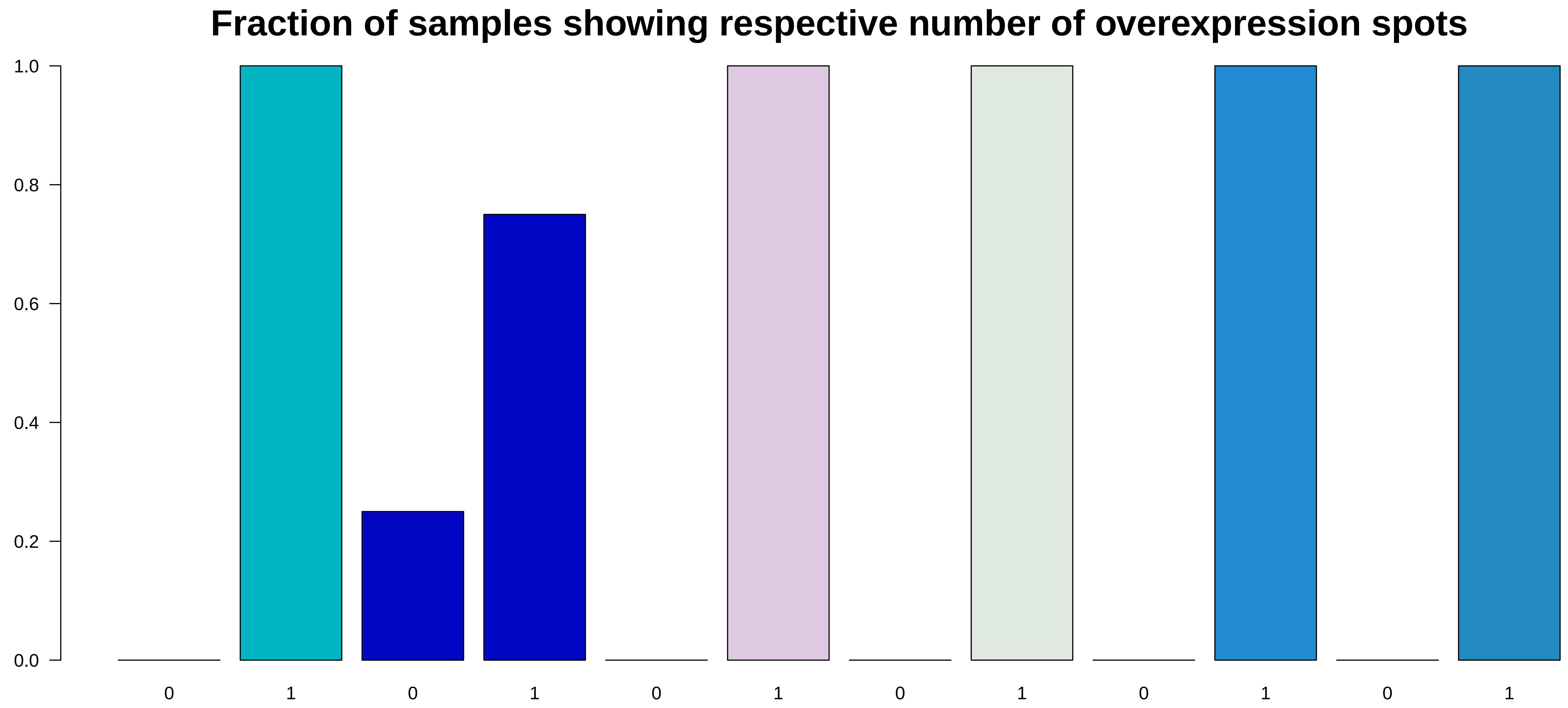
Fraction of samples showing respective number of overexpression spots
1.0
0.8
0.6
0.4
0.2
0.0
0
1
0
1
0
1
0
1
0
1
0
1

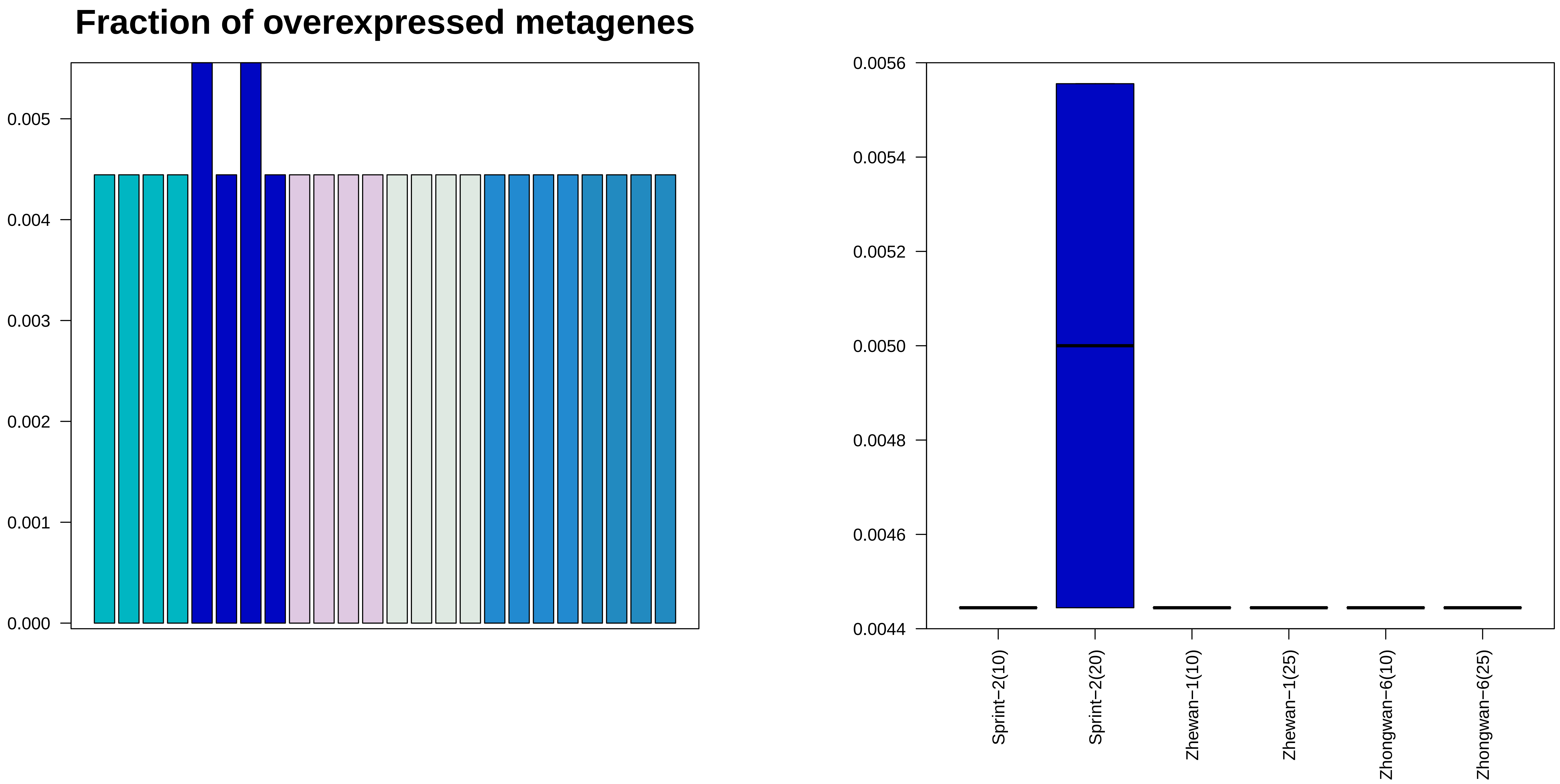

Fraction of overexpressed metagenes
0.000
0.001
0.002
0.003
0.004
0.005
0.0044
0.0046
0.0048
0.0050
0.0052
0.0054
0.0056
Sprint−2(10)
Sprint−2(20)
Zhewan−1(10)
Zhewan−1(25)
Zhongwan−6(10)
Zhongwan−6(25)

Length of borderline along overexpressed metagenes

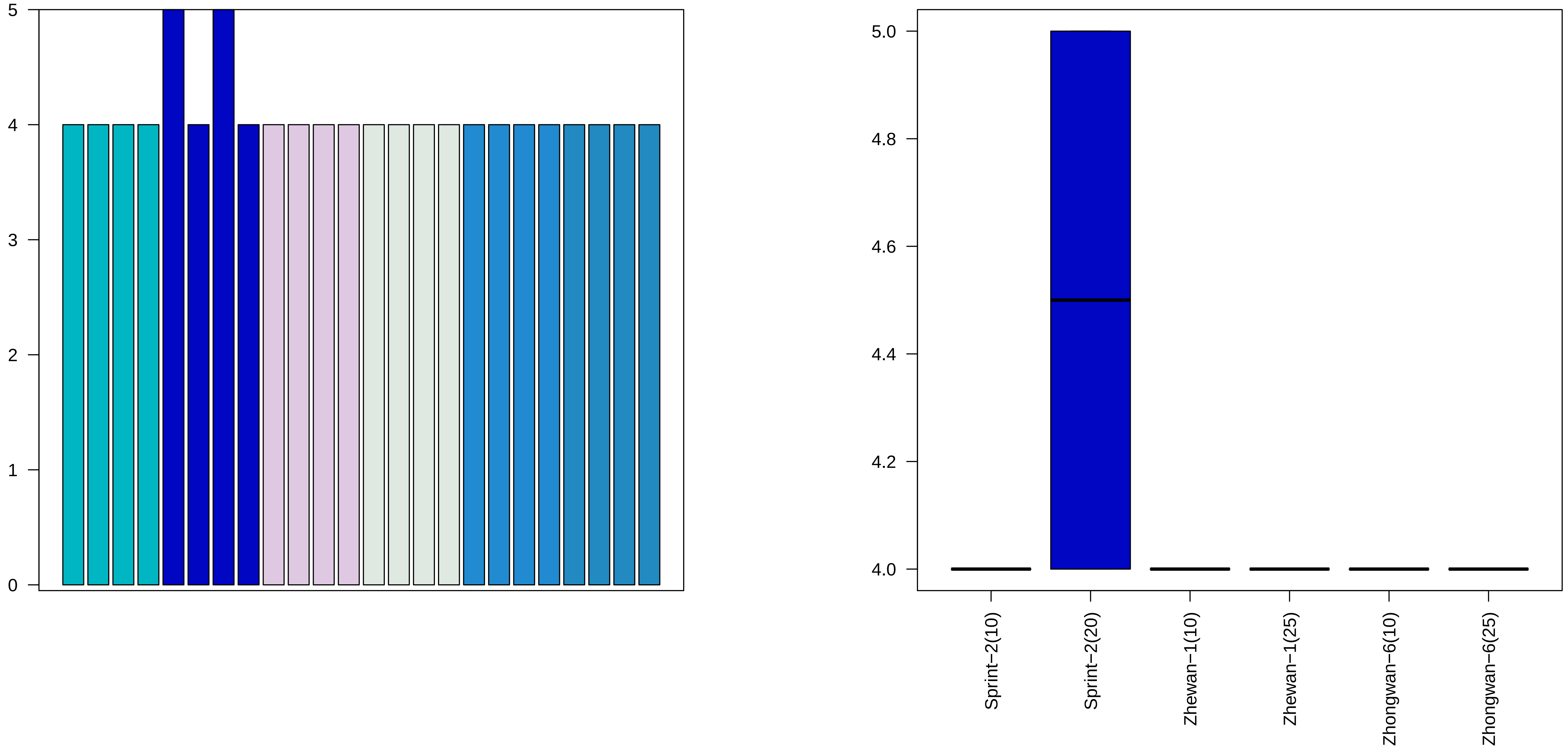

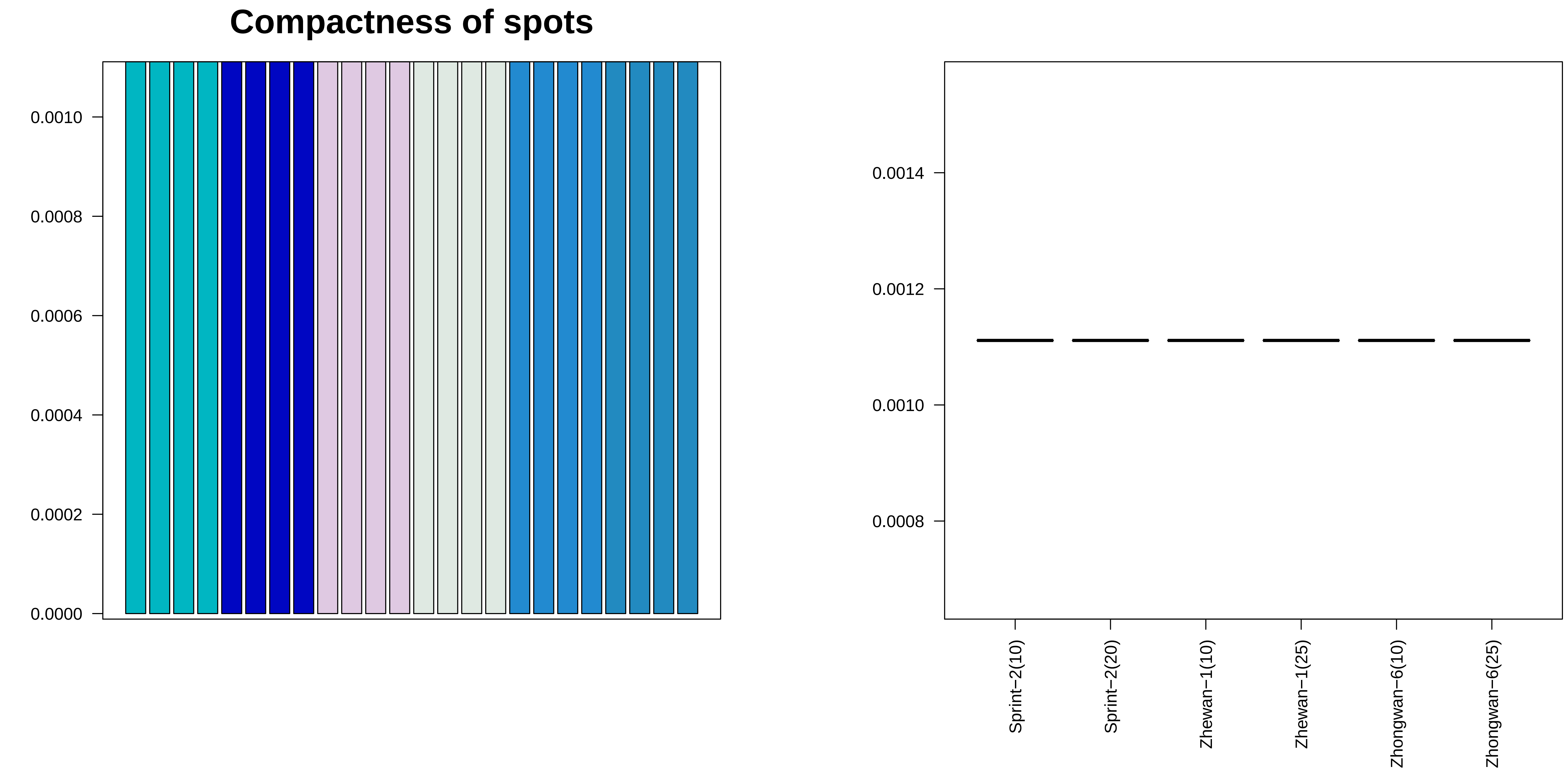

Compactness of spots
0.0010
0.0008
0.0006
0.0004
0.0002
0.0000
0.0014
0.0012
0.0010
0.0008
Sprint−2(10)
Sprint−2(20)
Zhewan−1(10)
Zhewan−1(25)
Zhongwan−6(10)
Zhongwan−6(25)

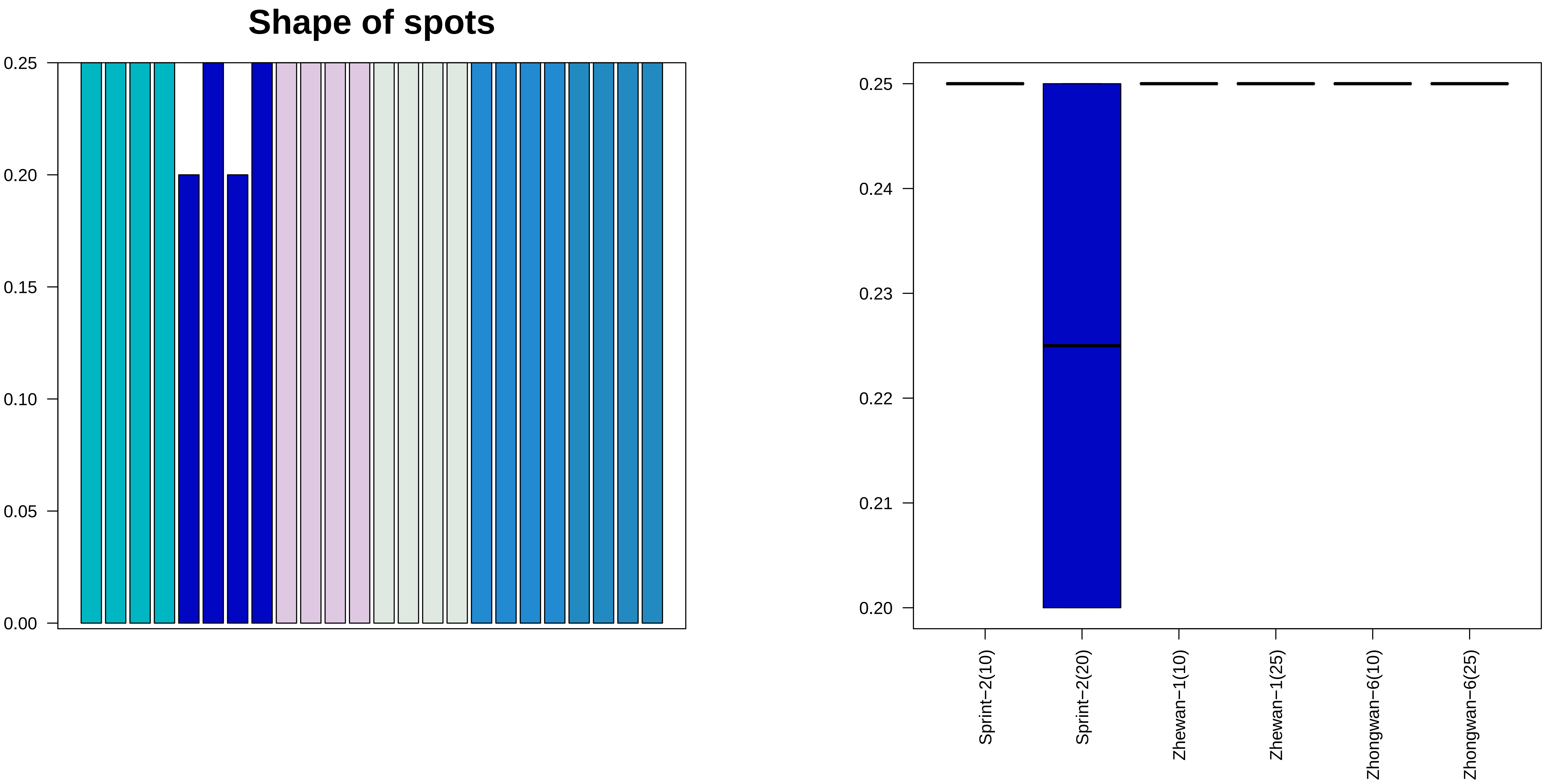
Shape of spots
0.25
0.20
0.15
0.10
0.05
0.00
0.25
0.24
0.23
0.22
0.21
0.20
Sprint−2(10)
Sprint−2(20)
Zhewan−1(10)
Zhewan−1(25)
Zhongwan−6(10)
Zhongwan−6(25)
